# Annotations

**Published:** 1901-09-07

**Authors:** 


### ANNOTATIONS.

### Railway Servants and Nervous Strain.

DR. SCOTT, assistant physician to the Glasgow Royal Infirmary, says that the neuroses of railway servants form an alarming phase of the dangerous character of this occupation. He refers more particularly to engine drivers and signalmen, and holds that many accidents, the majority in fact, are due to the nervous tension at which these men are kept while performing their duties—a tension which only few of them are constituted to withstand. He gives several cases in point, which go to show that men who may be perfectly fit for ordinary occupation sometimes utterly break down under the nervous strain of responsible railway work. One in particular we may mention, because it is a type of a whole class of digestive disorders arising from nervous strain, from whatever cause this may arise, cases which nothing cures except removal of the cause. "A man who had been a stoker was promoted to the position of engine-driver. This man some time after came and consulted him with all the symptoms of apparent atonic dyspepsia. All the usual remedies failed to relieve him, and in consequence he was sent to a consulting physician, who was also unsuccessful. Then the unfortunate man went through the whole course of quacks in the town without success. It was only after a big smash on the railway on which the man was dismissed that he finally got quite well. It was then evident that the man had simply been suffering from nervous tension. He had been well ever since." Of course the same applies to many other forms of occupation besides those connected with railways. We doubt not that we could find in the City of London many a clerk broken down by dyspepsia and a whole string of neuroses, even to madness, due purely to the ever present and overpowering sense of responsibility—the nervous tension—involved in their work. We have seen the same thing in mechanics, in cabinetmakers, and even in dressmakers when set to work upon valuable material, so that probably the neuroses of railway servants are but peculiar in their intensity, which arises from the constant sense of the appalling nature of the catastrophe which would result from any error. Turning from railways to electric cars Dr. Scott very properly points out the extreme condition of nervous tension involved in driving them, and certainly when these are made to spin along at a high speed, with children popping out at every corner, the strain is constant and very trying.

### The True Medical Missionary.

No one can read the eloquent speeches made by Sir William MacGregor and Major Donald Ross, both medical men, on the occasion of the visit of the latter to Lagos, or consider the details therein given regarding the work which is now being carried out in that Colony, with the object of ridding it and the great hinterland, to which it is the natural port, of that malarial infection which has hitherto stood in the way of its material progress, without admitting that the true medical missionary is he who preaches the doctrine of health and endeavours to carry into effect those measures which modern medical knowledge shows to be most effectual in the prevention of disease. Medicine when popularised too often becomes mere twaddle, old woman's talk served up with a few pseudo-scientific phrases to give it a savour of modernity. Sanitation, on the other hand, must be popular if it is to be useful. In the treatment of actual disease expert knowledge, skill, and training are required. Hence the doctor. But in health a man must himself do what is necessary to keep well. Thus it is essential that the knowledge of sanitary matters should be widespread; and the dissemination of this knowledge is the work of that sort of medical missionary by whom those demons malaria and typhoid fever are to be attacked and overthrown. Think of the condition described by Sir William MacGregor. A British Colony, a fertile country, occupied by 20 millions of people—people who have shown themselves capable of attaining a high degree of civilisation—a country which is being carefully administered, in which forest reserves are being set apart, roads are being made, railroads are being constructed, model farms are being opened, the people are being educated in industrial pursuits, and with the interior of which there is every prospect of a great and mutually advantageous trade; and all held back, all progress hindered, all faith in the country undermined, because of one thing, namely, fever. Can we wonder then that when the people of Lagos see what is being done in their midst and in Sierra Leone to rid them of this pestilence, they should welcome Major Ross as a true medical missionary.

### The Royal Commission on Tuberculosis.

ROYAL Commissions are not always very satisfactory machines for the discovery of truth. An excessive desire to be impartial is apt to make their pronouncements flabby, and not infrequently—perhaps with the object of giving their decisions weight in the eyes of the public—their members are selected for their names rather than for their knowledge of the subject in hand. The Royal Commission on Tuberculosis which has just been appointed is, however, altogether satisfactory. Sir Michael Foster, F.R.S., Professor of Physiology in the University of Cambridge; Dr. Sims Woodhead, Professor of Pathology in the University of Cambridge; Dr. Sidney Martin, Professor of Pathology at University College, London; Dr. John McFadyean, Principal and Professor of Comparative Pathology and Bacteriology at the Royal Veterinary College; and Professor Boyce, of University College, Liverpool, form a group of men of world-wide fame, whose knowledge is undoubted, whose powers of investigation are unsurpassed, and who have among them just that dash of difference between their several opinions and points of view which suffices to give confidence that the subject of their investigations is not a *chose jugée* The terms of the reference, which are fairly complete, are as follows: To inquire and report with respect to tuberculosis "1. Whether the disease in animals and in man is one and the same. 2. Whether animals and man can be reciprocally infected with it. 3. Under what conditions, if at all, the transmission of the disease from animals to man takes place, and what are the circumstances favourable or unfavourable to such transmission." We may opine that a good deal of latitude will have to be allowed in regard to the first point. The diseases are obviously different; the question is whether the infective cause is the same. It is also to be noted that no reference is made to the conditions favourable to the transmission of the disease from man to animals, which is perhaps a pity, for it is just within the bounds of possibility that it may turn out with regard to the tuberculosis bacillus as with some other microscopic forms of life, that a double cycle may be necessary for its continued existence, and that although it may be easily propagated from cow to cow or from man to man, an occasional transference from one species to another may be necessary for its continued existence.

